# AQP4 antibody-induced astrocyte injury reshapes CNS immune architecture in a combined EAE–AQP4-IgG model of neuromyelitis optica spectrum disorder

**DOI:** 10.3389/fimmu.2026.1899823

**Published:** 2026-10-19

**Authors:** Donghun Hyun, Junhyung Myung, Jin-Woo Park, Wen Li Leow, Yongwon Cho, Hyeonji Lee, Hyerin Lee, Jaewoong Lee, Byung-Jo Kim, Sunghoi Hong

**Affiliations:** 1 Department of Integrated Biomedical and Life Science, Korea University, Seoul, Republic of Korea; 2 BK21 FOUR Institute of Precision Public Health, Korea University, Seoul, Republic of Korea; 3 Department of Neurology, Korea University Anam Hospital, Korea University Medicine, Seoul, Republic of Korea

**Keywords:** aquaporin-4 (AQP4), astrocytopathy, experimental autoimmune encephalomyelitis (EAE), human complement, multiple sclerosis (MS), neuromyelitis optica spectrum disorder (NMOSD)

## Abstract

Neuromyelitis optica spectrum disorder (NMOSD) and multiple sclerosis (MS) are inflammatory autoimmune diseases of the central nervous system (CNS) that share demyelinating features but differ fundamentally in pathogenesis. The discovery of neuromyelitis optica immunoglobulin G antibodies (NMO-IgG) that target aquaporin-4 (AQP4) established NMOSD as a distinct clinical entity, although the experimental platforms used to dissect the mechanistic basis of this disorder remain insufficiently defined. Thus, this study aimed to develop a combined EAE-AQP4-IgG model by intrathecally delivering patient-derived NMO-IgG and human complement into pre-immunized mice to compare the associated pathology with that of experimental autoimmune encephalomyelitis (EAE), a representative model of MS. Both models exhibited blood–brain barrier (BBB) disruption, reflected by altered ZO-1 and CD31 expression. Notably, glial fibrillary acidic protein (GFAP) was strongly upregulated in EAE, whereas in the combined EAE-AQP4-IgG model, GFAP was acutely lost, indicating antibody-mediated astrocyte destruction. Consistently, AQP4 expression was markedly reduced in combined EAE-AQP4-IgG model animals. Combined EAE-AQP4-IgG model mice also displayed more severe demyelination and inflammation, confirmed by Luxol Fast Blue and H&E staining, electron microscopy, and MBP/Iba1 immunostaining. Quantitative reverse transcription polymerase chain reaction (qRT-PCR) further revealed distinct inflammatory signatures: *CCL2* and *BCL6* were elevated in EAE, whereas *IL-6*, *IL-17A*, *CXCL13*, and *PRDM1* were strongly upregulated in combined EAE-AQP4-IgG model. These differences were consistently observed across the brain, spinal cord, and optic nerve, albeit with regional variation. These results suggest that AQP4-IgG-induced astrocyte injury may redirect the CNS-related inflammatory milieu, shifting the CCL2-dominant, repair-permissive EAE environment into an IL-6–CXCL13–PRDM1 axis-driven, B cell-amplifying, remyelination-inefficient state that closely resembles the immunopathological context of human NMOSD. This combined EAE-AQP4-IgG model provides a robust platform for mechanistic investigation and therapeutic development.

## Introduction

Neuromyelitis optica spectrum disorder (NMOSD) and multiple sclerosis (MS) are distinct inflammatory demyelinating diseases of the central nervous system (CNS) that have historically been regarded as a single condition due to overlapping clinical and radiological features (1–5). Both disorders lead to severe neurological deficits through immune-mediated damage to myelin and glial cells. However, recent immunopathological discoveries have revealed fundamental differences in the pathogenesis, cellular targets, and underlying disease mechanisms of these conditions (1–3). Moreover, the identification of serum autoantibodies targeting aquaporin-4 (AQP4-IgG), a water channel expressed on astrocytic endfeet, has established NMOSD as a primary astrocytopathy distinct from the oligodendrocyte-targeted demyelination characteristic of MS (6–11).

In NMOSD, AQP4-IgG binding to astrocytes triggers complement-dependent cytotoxicity and secondary neuroinflammatory cascades, leading to astrocyte destruction, blood–brain barrier (BBB) disruption, and demyelination (6–11). Clinically, NMOSD manifests with severe optic neuritis and longitudinally extensive transverse myelitis, while each attack often results in irreversible disability (2, 8, 11, 12). Conversely, MS is primarily driven by autoreactive T cells and macrophage infiltration, which induce myelin loss and axonal injury, but typically allows for partial remyelination during remission (1, 13–15). These immunopathological distinctions underline the need for disease-specific models to elucidate mechanisms and evaluate targeted therapies (1, 3, 4, 13, 14).

Animal models play a crucial role in elucidating the pathogenesis of NMOSD and in developing novel therapeutic strategies (10, 16, 17). While the EAE model effectively recapitulates T cell-driven demyelination as a representative model of MS, reproducing the astrocytopathic features unique to NMOSD has proven more challenging (10, 11, 16). Although multiple NMOSD modeling strategies have been developed — including passive transfer of patient-derived AQP4-IgG, focal antibody delivery, and transgenic approaches — many earlier models failed to induce robust astrocyte loss due to species-specific incompatibility between human AQP4-IgG and the rodent complement system (17–19). Recent advances have therefore emphasized combining patient-derived AQP4-IgG with exogenous human complement to faithfully activate the complement cascade and reproducibly destroy astrocytes *in vivo* (19–22). Building on this approach, we hypothesized that AQP4-IgG–mediated astrocyte injury is not merely a structural endpoint but a critical initiating event that reshapes the local CNS immune microenvironment, driving immune profiles distinct from those observed in MS and contributing to the severe and frequently irreversible clinical course of NMOSD (8, 23).

Thus, this study aimed to develop and optimize a combined EAE-AQP4-IgG model by administering patient-derived NMO-IgG and exogenous human complement in pre-immunized EAE mice (10, 11). This approach will enable comparison of histological and molecular characteristics between NMOSD and MS models within the same experimental framework (4, 10, 14). Comprehensive analyses of markers associated with astrocytes (glial fibrillary acidic protein (GFAP), AQP4), BBB integrity (ZO-1, CD31), immune modulators (IL-1β, IL-6, TNF-α, CCL2, IL-17A, CXCL13, PRDM1, and BCL6), myelin-related genes (*MBP*, *Olig2*, *FGF17*), and astrocyte-secreted factors (PDGFα, PDGFβ, and CNTF), revealed distinct pathological signatures that parallel clinical observations in human NMOSD and MS (7, 8, 10, 11, 24). Our combined EAE-AQP4-IgG model generated through intrathecal administration of patient-derived NMO-IgG and exogenous human complement into EAE mice closely reproduced hallmark pathological features observed in NMOSD patients, including the loss of AQP4 and GFAP, complement-mediated astrocyte destruction, BBB disruption, microglial activation, and extensive demyelination in the brain, spinal cord, and optic nerve. These results demonstrate that AQP4-IgG-induced astrocytopathy redirects CNS immune signatures toward a non-regenerative, plasma cell-biased state in NMOSD, thereby bridging experimental modeling and clinical translation in NMOSD pathogenesis and therapeutic development.

## Results

### Behavioral evaluation of the combined EAE-AQP4-IgG model animals constructed through patient NMO-IgG injection

To construct the combined EAE-AQP4-IgG model, the female C57BL/6J mice were first immunized with MOG_35–55_ using complete Freund’s adjuvant (CFA) at day 0 and then injected with pertussis toxin (PTX) on days 0 and 1. Mice in the AQP4-IgG-H and AQP4-IgG-L groups received 500 g and 200 g of NMO-IgG, respectively together with human complement via intrathecal injection on days 8 and 11 post-immunization (**Figure 1A**).

**Figure 1:**
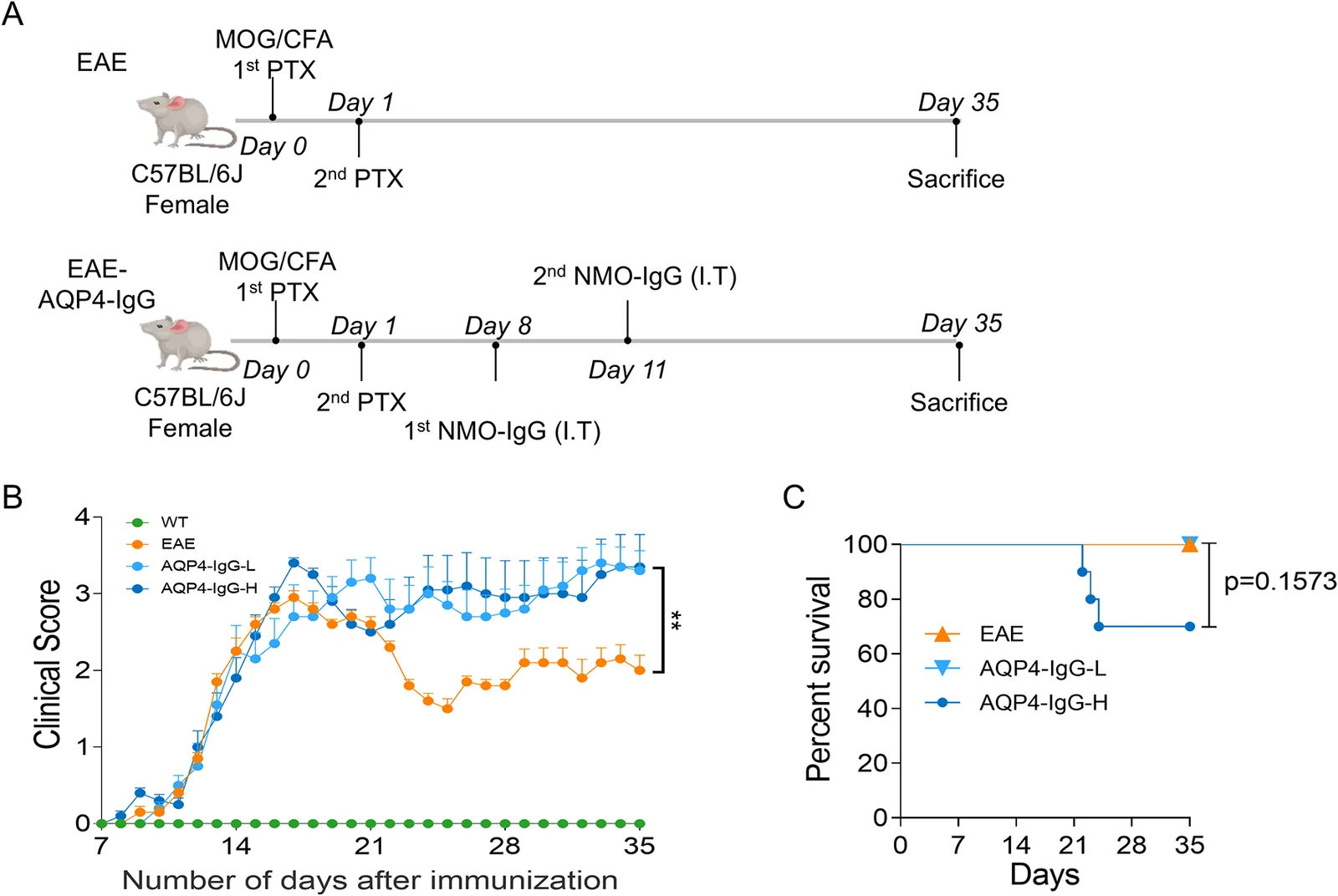
Induction and clinical characteristics of the combined EAE-AQP4-IgG model. **(A)** Schematic of combined EAE-AQP4-IgG model induction. **(B)** Clinical scoring showing more severe neurological deficits in AQP4-IgG-L and AQP4-IgG-H groups than EAE, with no significant difference between dose groups. **(C)** Survival curve demonstrating dose-dependent toxicity in the AQP4-IgG-H group; AQP4-IgG-L was used for subsequent analyses. n = 10 per group.

The cytotoxicity of the patient-derived NMO-IgG was validated *in vitro* using human astrocytes (HAs) before *in vivo* administration. Indeed, when HAs were exposed to varying concentrations of NMO-IgG in the presence of exogenous human complement, dose-dependent cytotoxicity was observed, indicating that these antibodies induce complement-mediated astrocyte injury (**Supplementary Figures 1A–C**).

The EAE clinical scores were recorded daily for 35 days post-immunization. The combined EAE-AQP4-IgG model groups receiving AQP4-IgG-L or AQP4-IgG-H exhibited more severe symptoms and sustained deficits, whereas the EAE mice, serving as controls, showed progressive neurological symptoms that plateaued after day 17. No significant differences were observed in the EAE scores between the AQP4-IgG-L and AQP4-IgG-H groups, indicating that even a low AQP4-IgG dose is sufficient to construct combined EAE-AQP4-IgG model animals in our experiments (**Figure 1B**). Meanwhile, mortality in our survival analysis was observed only in the AQP4-IgG-H group (**Figure 1C**), suggesting that excessive AQP4-IgG administration may cause systemic toxicity or acute adverse effects in addition to functional neurological impairment.

These results suggest that combined EAE-AQP4-IgG models with the optimal NMO-IgG concentration, achieved by intrathecal administration, may be required for studies of NMOSD, including pathogenic mechanisms and treatments.

### Complement-mediated astrocytopathy evidenced by C5b-9 deposition, loss of AQP4 and GFAP, and BBB destruction in the combined EAE-AQP4-IgG model

To examine whether the NMO-IgG causes astrocytopathy in the combined EAE-AQP4-IgG model animals, mice were sacrificed at day 35, and the brains, spinal cords, and optic nerves were harvested and stained with GFAP and AQP4 antibodies. The immunofluorescence assays revealed distinct patterns of GFAP and AQP4 immunostaining across the WT, EAE, AQP4-IgG-L, and AQP4-IgG-H model groups. The number and immunostained area of the GFAP^+^ cells in the EAE model were significantly increased compared to the WT, whereas the AQP4+ area was similar to that of the WT. These results indicate that the EAE model animals exhibit reactive astrogliosis and astrocyte hypertrophy across all brain tissues, the spinal cord, and the optic nerves, which are consistent with previous reports (25, 26). However, the combined EAE-AQP4-IgG model animals receiving AQP4-IgG-L or AQP4-IgG-H demonstrated a remarkable reduction in GFAP^+^ cell numbers and in GFAP^+^ and AQP4^+^ areas across all regions of the brain, spinal cord, and optic nerve tissues compared with the WT and EAE animals, respectively (**Figures 2A–F**). Quantitative reverse transcription polymerase chain reaction (qRT-PCR) analysis revealed that the expression levels of *GFAP* and *AQP4* were significantly increased in the EAE model compared with WT, but markedly reduced in the combined EAE-AQP4-IgG model relative to EAE (**Figures 2G–I**). These results show widespread astrocytopathy, as evidenced by the loss of AQP4 and GFAP, in combined EAE-AQP4-IgG model animals induced by NMO-IgG and complement, consistent with a previous report (20).

**Figure 2:**
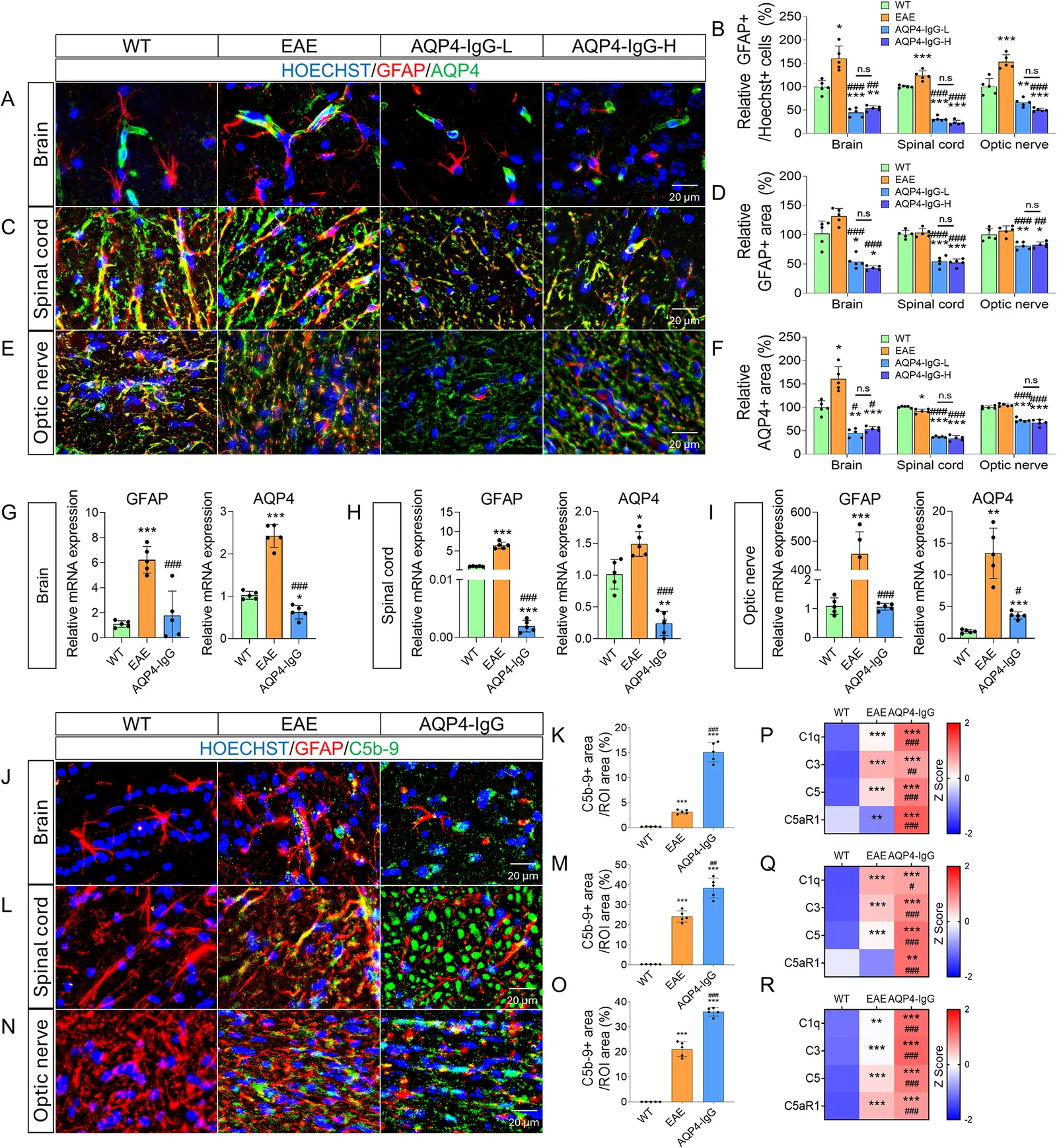
Astrocyte loss and AQP4 depletion in combined EAE-AQP4-IgG model. **(A, C, E)** Immunofluorescence images of GFAP and AQP4 in brain, spinal cord, and optic nerve. **(B, D, F)** Quantification showing significant reductions in GFAP^+^ cell numbers and GFAP^+^, AQP4^+^ area in combined EAE-AQP4-IgG model versus EAE and WT. **(G–I)** qRT-PCR confirming GFAP and AQP4 upregulation in EAE but marked downregulation in combined EAE-AQP4-IgG model. **(J, L, N)** Representative GFAP (red)/C5b-9 (green)/Hoechst (blue) double-immunofluorescence images of brain **(J)**, spinal cord **(L)**, and optic nerve **(N)** at Day 13. **(K, M, O)** Quantification of C5b-9-positive area in brain **(K)**, spinal cord **(M)**, and optic nerve **(O)**. **(P–R)** Heatmaps of complement genes (C1q, C3, C5, and C5aR1) in brain **(P)**, spinal cord **(Q)**, and optic nerve **(R)**. Heatmap values are presented as mean gene-wise Z-scores derived from log2-transformed expression values. Data: mean ± SD; *P < 0.05, **P < 0.01, ***P < 0.001 vs. WT; ^#^P < 0.05, ^##^P < 0.01, ^###^P < 0.001 vs. EAE.

To directly confirm complement activation and deposition at sites of astrocyte injury *in vivo*, we performed immunofluorescence staining for C5b-9 (membrane attack complex, MAC) on brain, spinal cord, and optic nerve sections from WT, EAE, and combined EAE-AQP4-IgG model mice. C5b-9 deposits were absent in WT tissues and only weakly detected in EAE, whereas prominent C5b-9 accumulation was observed throughout all three CNS regions in the combined EAE-AQP4-IgG model (**Figures 2J–O**). To further characterize the complement cascade at the transcriptional level, qRT-PCR analysis of C1q, C3, C5, and C5aR1 demonstrated significant upregulation of all four complement genes in the brain, spinal cord, and optic nerve of combined EAE-AQP4-IgG model animals compared with both WT and EAE controls (**Figures 2P–R**; **Supplementary Figures 4A–C**). Together, these findings provide direct *in vivo* evidence that intrathecally delivered human complement is activated and deposited at astrocyte injury sites, confirming complement-dependent cytotoxicity as the primary mechanism underlying AQP4-IgG–mediated astrocytopathy in our model.

Next, to examine whether NMO-IgG causes BBB disruption in combined EAE-AQP4-IgG models, brain, spinal cord, and optic nerve tissues were stained with a ZO-1 antibody to assess tight junctions and a CD31 antibody to evaluate the endothelial cells that comprise the barriers. The immunostaining patterns with the ZO-1 and CD31 antibodies were clearly different across the animal models. The immunostaining signals for ZO1, but not CD31, mostly disappeared in each tissue type of the EAE and combined EAE-AQP4-IgG models compared to WT, and the colocalized area between ZO1- and CD31-positive signals was dramatically decreased in the EAE and combined EAE-AQP4-IgG models. Interestingly, the colocalized area between the ZO1- and CD31-positive signals in the combined EAE-AQP4-IgG model animals was significantly reduced compared with those of EAE (**Figures 3A–F**), indicating that BBB disruption was more severe in the combined EAE-AQP4-IgG model than in the EAE model; these findings are consistent with previous clinical and experimental results showing that AQP4-IgG-mediated astrocyte injury leads to profound BBB breakdown in NMOSD (27, 28).

**Figure 3:**
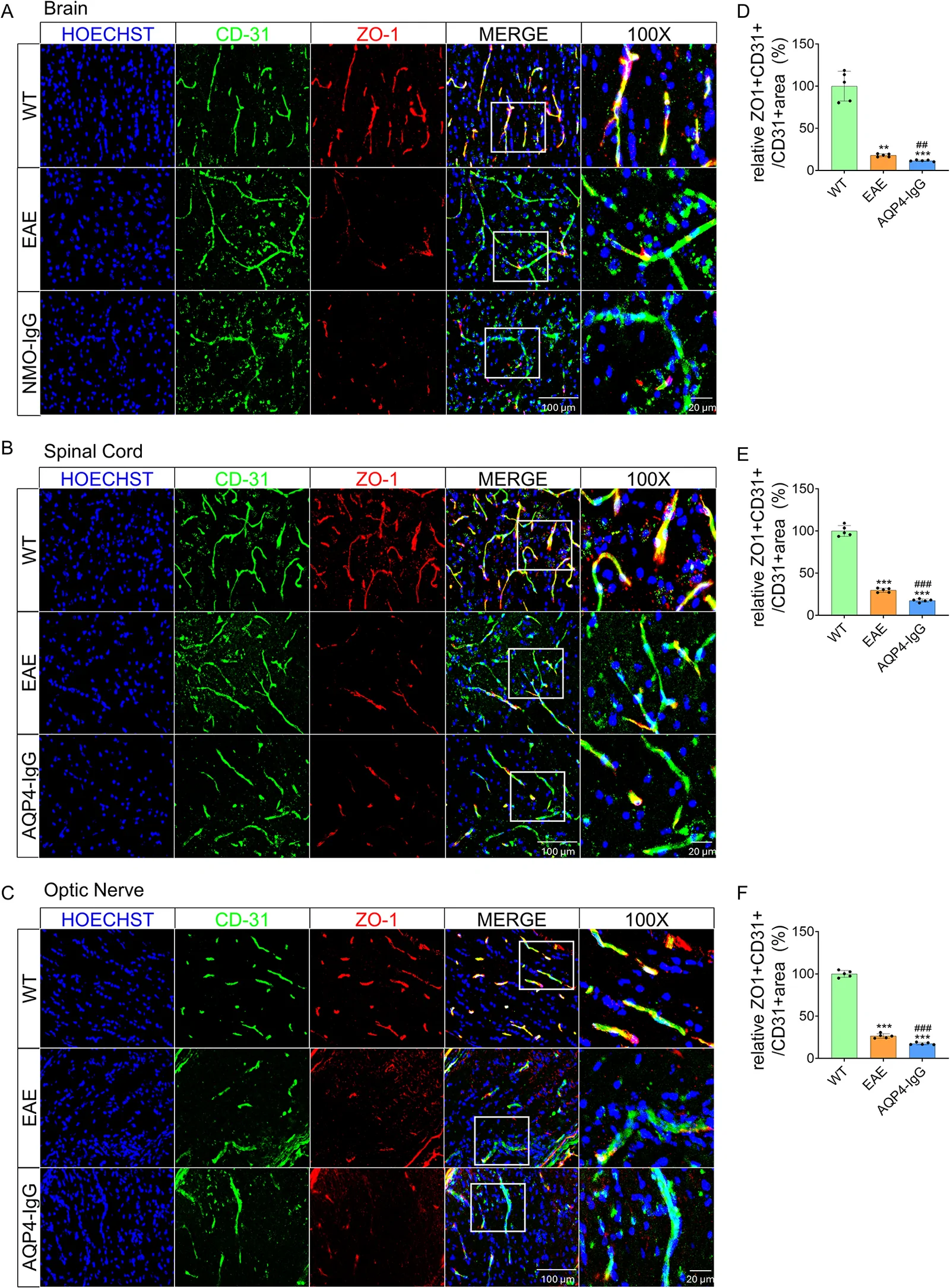
BBB impairment in the EAE and combined EAE-AQP4-IgG models. **(A–C)** Immunofluorescence images of ZO-1 and CD31 in brain, spinal cord, and optic nerve. **(D–F)** Quantification of ZO-1/CD31 colocalized area showing significantly greater BBB disruption in combined EAE-AQP4-IgG model than in EAE across all CNS regions. Data: mean ± SD; *P < 0.05, **P < 0.01, ***P < 0.001 vs. WT; ^#^P < 0.05, ^##^P < 0.01, ^###^P < 0.001 vs. EAE.

Therefore, these data suggest that a low dose of NMO-IgG is suitable to induce a combined EAE-AQP4-IgG model in the current study and for future investigations.

### A coordinated transcriptional and cellular network underlies the Th17–B cell inflammatory signature in the combined EAE-AQP4-IgG model

Next, to examine the inflammatory responses in the combined EAE-AQP4-IgG model animals, we performed H&E staining and qRT-PCR on brain, spinal cord, and optic nerve tissues harvested from WT, EAE, and combined EAE-AQP4-IgG model mice. Our histological analysis showed no inflammation in WT mice, whereas the disease model animals exhibited substantial immune cell infiltration across all tissues (**Figures 4A–F**). Our semi-quantitative inflammation scoring indicated that the inflammatory responses were more pronounced in the combined EAE-AQP4-IgG model than in EAE.

**Figure 4:**
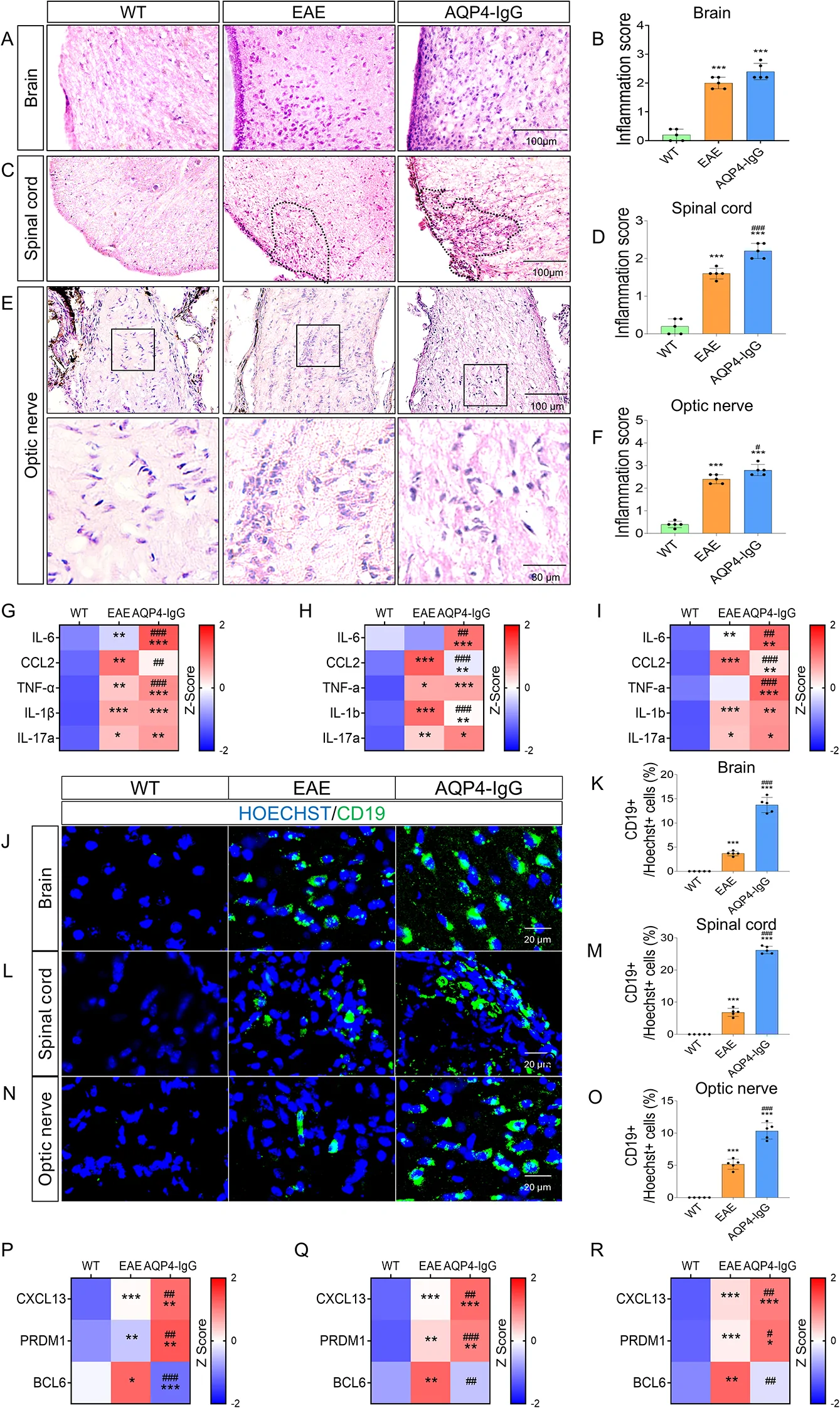
CNS region-specific inflammatory responses in the combined EAE-AQP4-IgG model. **(A, C, E)** Representative H&E images and **(B, D, F)** semiquantitative inflammation scores (0–3; 0–4 for optic nerve) of brain, spinal cord, and optic nerve. **(G–I)** Heatmaps of inflammatory cytokine and chemokine expression (IL-6, CCL2, TNF-α, IL-1β, IL-17A) by qRT-PCR, presented as mean gene-wise Z-scores derived from log2-transformed expression values. **(J, L, N)** Representative CD19 (green)/Hoechst (blue) immunofluorescence images and **(K, M, O)** quantification of CD19-positive cells relative to Hoechst-positive nuclei in brain, spinal cord, and optic nerve. **(P–R)** Heatmaps of CXCL13, PRDM1, and BCL6 expression by qRT-PCR, presented as mean gene-wise Z-scores derived from log2-transformed expression values. n = 5 mice per group. Data: mean ± SD; *P < 0.05, **P < 0.01, ***P < 0.001 vs. WT; #P < 0.05, ##P < 0.01, ###P < 0.001 vs. EAE.

Moreover, our immunostaining analysis exhibited increased numbers of microglia in both the EAE and combined EAE-AQP4-IgG models, accompanied by demyelination in the brains, spinal cords, and optic nerves compared to WT (**Supplementary Figures 2A, D, G**). The number of Iba1^+^cells was significantly increased in the EAE model compared with the WT, with an even greater increase observed in the combined EAE-AQP4-IgG model compared with the EAE model (**Supplementary Figures 2B, E, H**). The tissue intensity of the MBP antibody staining was significantly decreased in all the tissues from the combined EAE-AQP4-IgG model animals compared with EAE (**Supplementary Figures 2C, F, I**). These results demonstrate that optimized administration of AQP4-IgG in EAE model mice effectively established combined EAE-AQP4-IgG models, recapitulating the key pathological features observed in patients with NMOSD.

To further investigate the molecular signatures of adaptive immunity, particularly the distinct differentiation patterns of T cell, we analyzed the expression levels of genes, including *CCL2*, *IL-6*, *TNF-α*, *IL-1β*, *IL-17A*, *CXCL13* (**Figures 4G–I**; **Supplementary Figures 4D–F**). Interestingly, our gene expression analysis of the brain, spinal cord, and optic nerve tissues revealed that the inflammatory architecture of NMOSD is shaped not by isolated cytokine changes but by a coordinated reorganization of immune-regulatory networks. IL-6 was selectively and robustly induced in the combined EAE-AQP4-IgG model, indicating a secondary inflammatory cascade triggered by astrocyte injury and complement activation (29–32). The expression levels of *IL-17A* and *CXCL13* were increased in EAE relative to WT and were further elevated in the combined EAE-AQP4-IgG model. Together with the higher *IL-6* expression, these findings indicate an enhanced Th17- and B-cell-associated inflammatory transcriptional profile in the combined model relative to EAE. To further validate these inflammatory changes at the protein level, we performed immunofluorescence staining for IL-6 and CXCL13 in the brain, spinal cord, and optic nerve. Both IL-6- and CXCL13-positive areas were increased in EAE compared with WT and were further elevated in the combined EAE-AQP4-IgG model across all three CNS regions (**Supplementary Figures 3A–I**). These findings were consistent with the transcriptional changes observed by qRT-PCR. This result is consistent with the dominant Th17 axis and elevated *IL-17A* levels observed in the cerebrospinal fluid (CSF) of NMOSD patients relative to those of MS patients (33). CXCL13 upregulation promotes the recruitment of B cells and proinflammatory T cell subsets into the CNS, while the heightened Th17 signature further amplifies this chemokine-driven cellular influx. This distinction between the two models highlights the role of astrocytopathy in shaping the inflammatory landscape of NMOSD, distinguishing NMOSD from EAE-induced neuroinflammation. To directly validate B cell recruitment at the cellular level, we performed immunostaining for CD19, a pan-B cell marker, on brain, spinal cord, and optic nerve tissue sections from WT, EAE, and combined EAE-AQP4-IgG model mice. CD19^+^ cells were absent in WT tissues, whereas a modest increase was observed in EAE. Strikingly, CD19^+^ cell numbers were significantly elevated in the combined EAE-AQP4-IgG model compared with EAE across all three CNS regions (**Figures 4J–O**), providing cellular-level evidence of increased B cell infiltration, consistent with the elevated CXCL13 expression in this model. Our qRT-PCR analysis further showed that CXCL13 and PRDM1 expression were significantly increased, whereas BCL6 expression was significantly decreased, in the combined EAE-AQP4-IgG model compared with EAE across the brain, spinal cord, and optic nerve (**Figures 4P–R**; **Supplementary Figures 4G–I**).

Strikingly, these cytokine shifts converged on a distinct transcriptional program characterized by a PRDM1-high/BCL6-low state in NMOSD. This transcriptional configuration is consistent with a shift toward terminal B cell differentiation, potentially favored by the IL-6-rich milieu. In contrast, EAE maintained a BCL6-supported, germinal center-like regulatory balance aligned with the associated CCL2-dominant, repair-permissive inflammatory profile.

Together, these interconnected transcriptional and cytokine alterations indicate that astrocyte injury in combined EAE-AQP4-IgG model does not simply intensify inflammation but also reshapes the logic of adaptive immunity, consistent with a state that is distinct from the immune dynamics of EAE.

### Loss of myelin and molecular analyses in combined EAE-AQP4-IgG model animals

Next, to assess whether the combined EAE-AQP4-IgG model animals exhibit myelin loss, we performed Luxol Fast Blue (LFB) staining and qRT-PCR analyses. The LFB staining experiments showed a significant demyelination in the brain, spinal cord, and optic nerve tissues of the combined EAE-AQP4-IgG models. The intensity of LFB staining was significantly reduced in the tissues of the combined EAE-AQP4-IgG model animals compared with those of the EAE model, indicating a greater loss of myelin in the combined EAE-AQP4-IgG model animals (**Figures 5A–F**). Moreover, in our immunostaining analysis, both the EAE and combined EAE-AQP4-IgG models exhibited demyelination in the brains, spinal cords, and optic nerves of animals compared with WT controls (**Supplementary Figures 2A, D, G**). The tissue intensity stained with the MBP antibody was significantly decreased in all combined EAE-AQP4-IgG model tissues compared to EAE (**Supplementary Figures 2C, F, I**).

**Figure 5:**
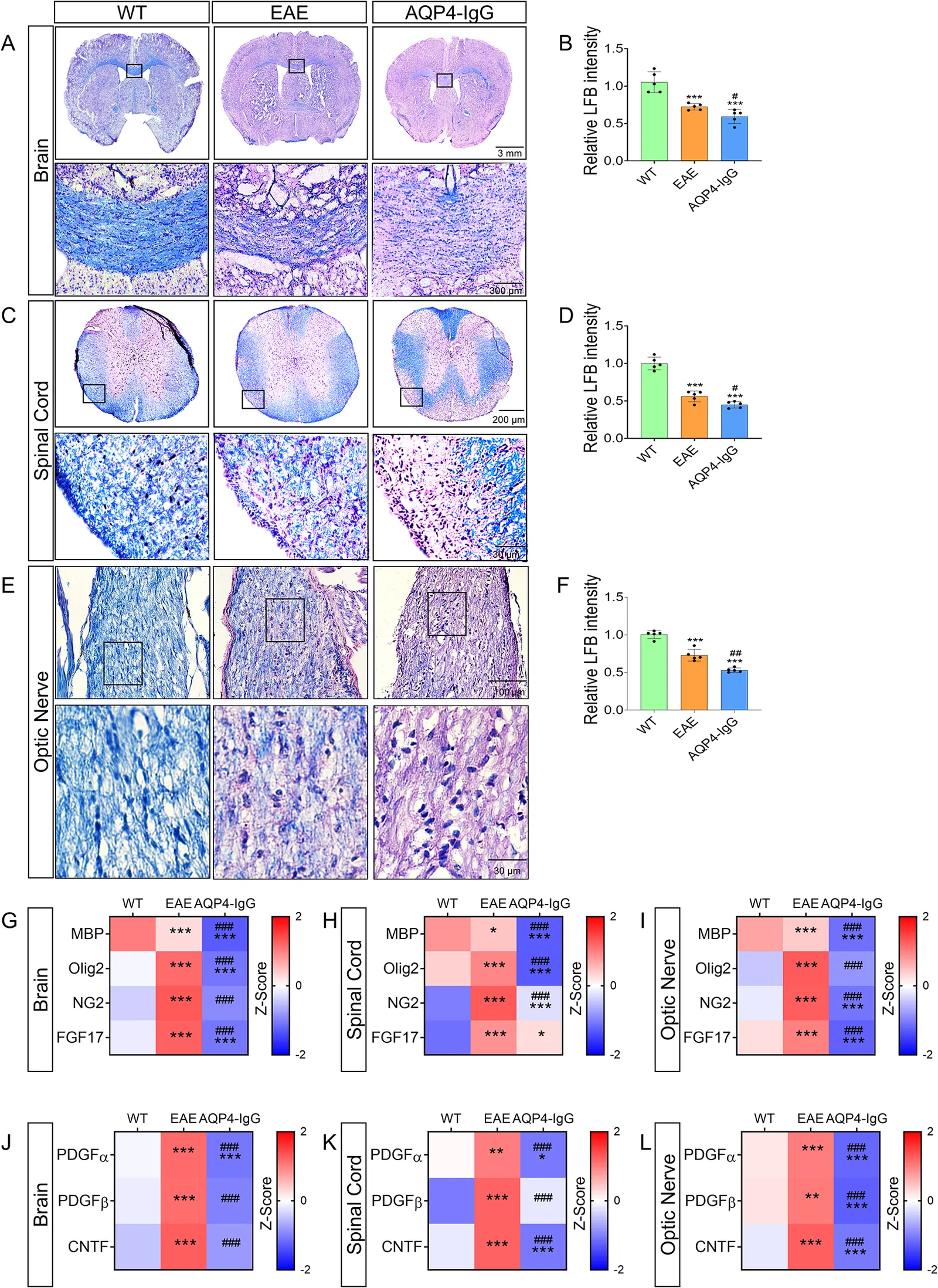
Differential demyelination and oligodendrocyte-associated gene expression in combined EAE-AQP4-IgG and EAE models. **(A, C, E)** LFB–H&E co-staining images of brain, spinal cord, and optic nerve. **(B, D, F)** Quantification of LFB intensity showing more severe demyelination in the combined EAE-AQP4-IgG model than EAE. **(G–I)** Heatmaps of qRT-PCR analysis of oligodendrocyte and OPC markers (MBP, Olig2, NG2, FGF17), showing upregulation in EAE but downregulation in the combined EAE-AQP4-IgG model. **(J–L)** Heatmaps of astrocyte-derived trophic factors (PDGFα, PDGFβ, CNTF), showing increased expression in EAE but decreased expression in the combined EAE–AQP4-IgG model, indicating a non-permissive environment for remyelination. Heatmap values are presented as mean gene-wise Z-scores derived from log2-transformed expression values. Data: mean ± SD; *P < 0.05, **P < 0.01, ***P < 0.001 vs. WT; ^#^P < 0.05, ^##^P < 0.01, ^###^P < 0.001 vs. EAE.

Next, we employed qRT-PCR to assess the expression levels of oligodendrogenesis-related genes, including *MBP*, *NG2*, *Olig2*, and *FGF17*, in the combined EAE-AQP4-IgG and EAE models. The expression levels of the *NG2*, *Olig2*, and *FGF17* genes—collectively known as oligodendrocyte precursor cell (OPC)-associated genes—as well as *MBP* as a well-established marker of mature oligodendrocytes, were significantly decreased in the brains, spinal cords, and optic nerves of combined EAE-AQP4-IgG model animals compared with those of EAE animals, indicating compromised myelin integrity (**Figures 5G–I**; **Supplementary Figures 4J–L**). Meanwhile, the *MBP*, *Olig2*, *NG2*, and *FGF17* gene expression levels were significantly increased in the brains, spinal cords, and optic nerves of the EAE model animals compared with those in the WT. Similar patterns have been reported in the brains of MS patients, in which increased oligodendrocyte precursor cell (OPC) markers are associated with reduced expression of myelin proteins (34).

Next, we utilized qRT-PCR to examine the expression levels of astrocyte-derived trophic factor genes. Specifically, *PDGFα*, *PDGFβ*, and *CNTF*, key astrocyte-secreted factors, play crucial roles in OPC proliferation, differentiation, and survival. Indeed, these trophic factors are critical for myelination and remyelination by facilitating the maturation of OPCs into myelinating oligodendrocytes, a process fundamental for CNS repair following demyelinating insults (14, 35). Our results showed that these factors were significantly downregulated in combined EAE-AQP4-IgG model animals, whereas the expression of these genes was notably upregulated in EAE model animals relative to WT (**Figures 5J–L**; **Supplementary Figures 4M–O**). These results suggest that astrocytopathy in NMOSD, caused by AQP4-IgG-mediated complement-dependent cytotoxicity, impairs the regenerative microenvironment necessary for OPC activation and remyelination. In contrast, EAE pathology typically involves astrocyte activation, which enhances trophic support during recovery phases. Importantly, these distinctions are consistent with clinical observations: astrocyte damage is an early and defining pathological feature of NMOSD with severe AQP4 loss and astrocyte destruction in patient lesions (24, 36, 37), whereas astrocytes are largely preserved and often become reactive in demyelinated regions in MS (38).

Therefore, these results show that the combined EAE-AQP4-IgG model exhibits distinct molecular and pathological characteristics compared to the EAE model. Although the two models show myelin loss in the brains, spinal cords, and optic nerves, differences in oligodendrogenesis-related gene expression and in the regulation of astrocyte-derived trophic factors suggest unique disease mechanisms. The combined EAE-AQP4-IgG model recapitulates several key features of astrocytopathy-driven demyelination observed in NMOSD patients, highlighting the potential of this model as a valuable tool for studying disease pathogenesis and therapeutic strategies.

### A profound structural remodeling of myelin architecture in the combined EAE-AQP4-IgG model

To further examine myelination states in the brains, spinal cords, and optic nerves of the two animal models, we conducted transmission electron microscopy (TEM) experiments on these tissues from WT, EAE, and combined EAE-AQP4-IgG model mice. The TEM imaging results showed marked disruption of myelin architecture in both disease models compared with the WT model (**Figures 6A, C, E**). Myelin sheaths appeared thinner and more disorganized in the EAE and combined EAE-AQP4-IgG models than in WT. Additionally, the characteristic dense lamellar compaction observed in healthy myelin was disrupted in the EAE and combined EAE-AQP4-IgG animal models. Interestingly, the myelin sheaths in the brains, spinal cords, and optic nerves of combined EAE-AQP4-IgG model mice showed significantly more structural abnormalities and appeared more wrinkled than in the EAE animal model. Myelin sheath thickness in the brains, spinal cords, and optic nerves of WT, EAE, and NMOSD was quantitatively measured and was significantly decreased in the EAE and NMOSD compared to the WT. However, axon diameter remained consistent across all groups, while the g-ratio was proportionally increased in the EAE and combined EAE-AQP4-IgG model animals (**Figures 6B, D, F**).

**Figure 6:**
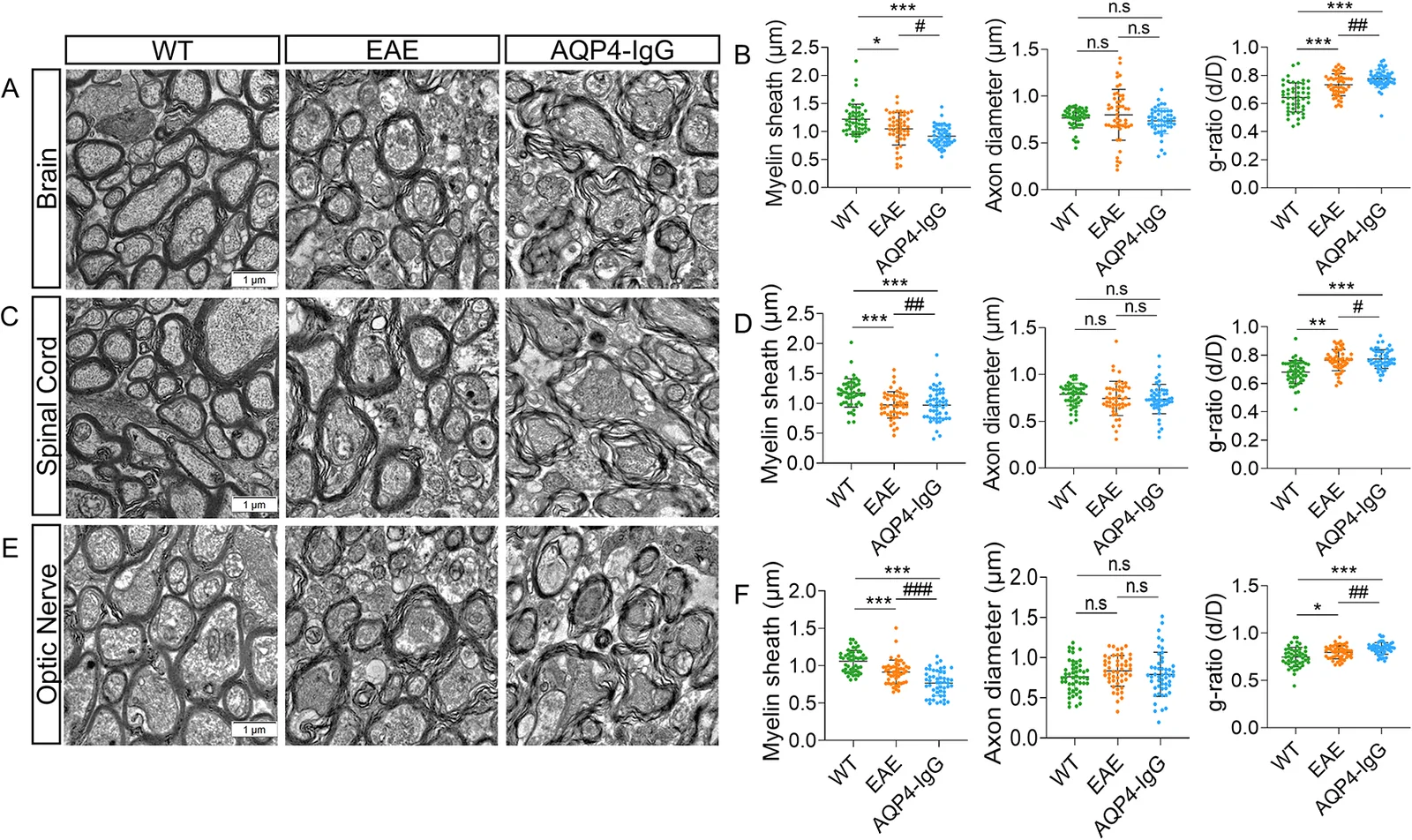
Disruption of ultrastructural myelin in the combined EAE-AQP4-IgG model. **(A, C, E)** TEM images of myelinated axons in brain, spinal cord, and optic nerve, showing progressive myelin disorganization in EAE and more severe wrinkling and lamellar disintegration in combined EAE-AQP4-IgG model. **(B, D, F)** Quantification of myelin sheath thickness, g-ratio, and axon diameter; sheath thickness is reduced and g-ratio increased in both models, most severely in combined EAE-AQP4-IgG model, while axon diameter is unchanged. n = 5 mice/group; 10 axons/mouse. Data: mean ± SD; *P < 0.05, **P < 0.01, ***P < 0.001 vs. WT; ^#^P < 0.05, ^##^P < 0.01, ^###^P < 0.001 vs. EAE.

These results demonstrate that the combined EAE–AQP4-IgG model exhibits extensive and pronounced demyelination, accompanied by profound myelin disruption without significant changes in axon diameter across the brain, spinal cord, and optic nerve. Collectively, these findings indicate that our combined EAE–AQP4-IgG model, distinct from EAE, provides a valuable platform for investigating NMOSD pathogenesis and therapeutic strategies.

### Temporal progression of complement activation, astrocyte loss, neuroinflammation, and demyelination across the disease course

To characterize the temporal evolution of pathological events and to determine whether complement deposition precedes astrocyte injury and downstream inflammatory changes, we performed analyses at Day 13 and Day 21 post-immunization as additional timepoints alongside the Day 35 endpoint.

At Day 13, immediately after the second intrathecal injection, C5b-9 MAC deposition and complement cascade activation were already evident across all CNS tissues of the combined EAE-AQP4-IgG model (**Figures 2J–R**; detailed above), establishing that complement activation is the earliest detectable event in the cascade. At Day 21, immunofluorescence staining for GFAP and AQP4 revealed significant reductions in astrocyte markers across all three CNS regions of the combined EAE-AQP4-IgG model (**Figures 7A–F**), confirming that complement-initiated astrocyte destruction is well established by this intermediate timepoint. H&E staining demonstrated substantially greater inflammatory cell infiltration in the combined EAE-AQP4-IgG model compared with EAE and WT at Day 21 (**Figures 7G–J**). Luxol Fast Blue staining further revealed progressive demyelination that was most severe in the combined EAE-AQP4-IgG model, intermediate in EAE, and minimal in WT, with all pairwise comparisons reaching statistical significance (**Figures 7K–N**). By Day 35, the early events had culminated in the fully established NMOSD-like phenotype characterized by sustained astrocyte loss, persistent BBB disruption, a coordinated Th17–B cell inflammatory axis, and progressive demyelination with impairment of remyelination capacity (as detailed in **Figures 2**–**6** above).

**Figure 7:**
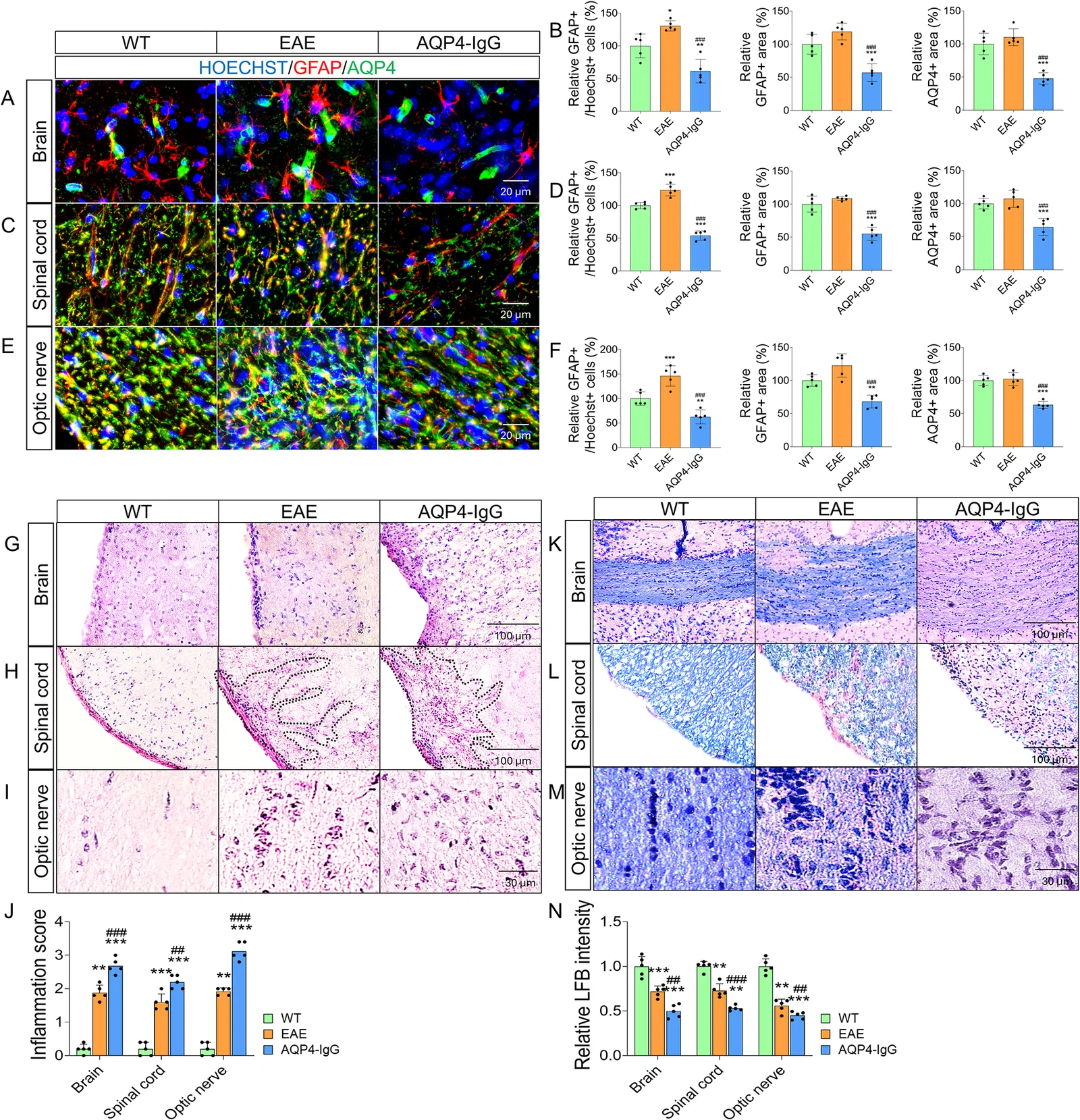
Temporal progression of complement activation, astrocyte loss, neuroinflammation, and demyelination in the combined EAE-AQP4-IgG model. **(A, C, E)** Representative GFAP/AQP4 double-immunofluorescence images of brain **(A)**, spinal cord **(C)**, and optic nerve **(E)** at Day 21. **(B, D, F)** Quantification of GFAP-positive cells, GFAP-positive area, and AQP4-positive area in brain **(B)**, spinal cord **(D)**, and optic nerve **(F)**. **(G–I)** Representative H&E images of brain **(G)**, spinal cord **(H)**, and optic nerve **(I)**, and **(J)** semiquantitative inflammation scores at Day 21. **(K–M)** Representative Luxol Fast Blue images of brain **(K)**, spinal cord **(L)**, and optic nerve **(M)**, and **(N)** quantification of staining intensity at Day 21. n = 5 mice per group. Data: mean ± SD; *P < 0.05, **P < 0.01, ***P < 0.001 vs. WT; ^#^P < 0.05, ^##^P < 0.01, ^###^P < 0.001 vs. EAE.

Together, the three-timepoint longitudinal analysis (Day 13 → Day 21 → Day 35) delineates a clear and mechanistically coherent pathological cascade: early complement activation and MAC deposition (Day 13) precedes measurable astrocyte loss and neuroinflammation (Day 21), which in turn drives progressive demyelination and immune architectural remodeling culminating at Day 35 (see schematic in **Figure 8**). This temporal sequence provides direct *in vivo* evidence that complement-dependent cytotoxicity is the initiating event in AQP4-IgG–mediated astrocytopathy and its downstream immunopathological consequences in the combined EAE-AQP4-IgG model.

**Figure 8:**
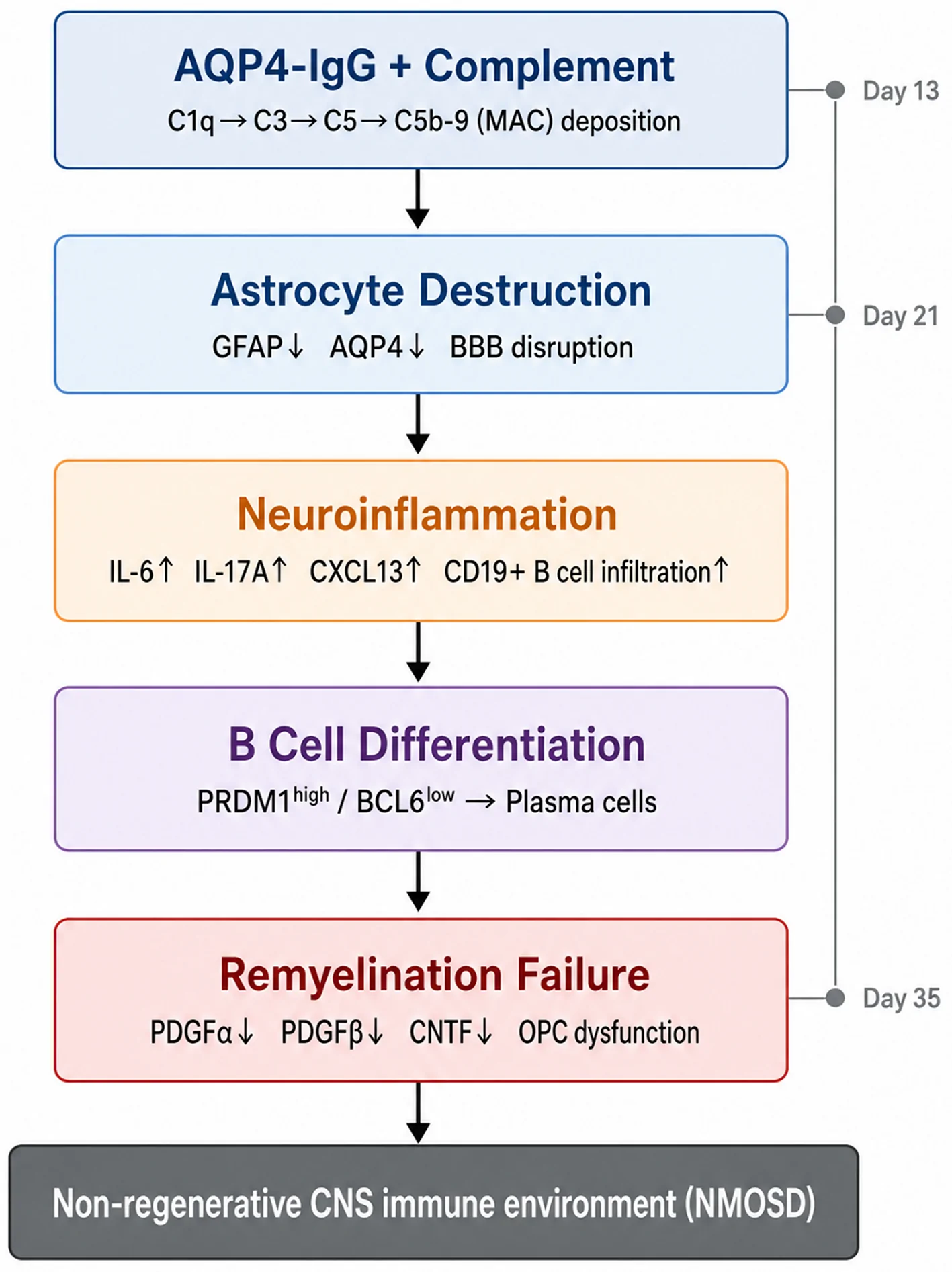
Schematic illustration of the proposed pathogenic cascade in the combined EAE-AQP4-IgG model. Intrathecal delivery of NMO-IgG and human complement initiates complement cascade activation and C5b-9/MAC deposition at sites of astrocyte injury (Day 13), followed by progressive astrocyte destruction (GFAP↓, AQP4↓) and BBB disruption (Day 21). The resulting IL-6 surge drives a self-reinforcing Th17–B cell axis characterized by IL-17A and CXCL13 upregulation and CD19^+^ B cell recruitment, converging on a PRDM1-high/BCL6-low transcriptional state that promotes plasma cell differentiation. Loss of astrocyte-derived trophic factors (PDGFα, PDGFβ, CNTF) and sustained microglial activation collectively establish a non-regenerative, B cell-amplifying CNS environment by Day 35, recapitulating key immunopathological features of human NMOSD.

## Materials and methods

### Astrocyte culture

Primary HAs (ScienCell, Cat#1800) were cultured in astrocyte medium (AM; ScienCell, Cat#1801) as per the manufacturer’s instructions. Culture flasks were pre-coated with 2 μg/cm² poly-L-lysine (ScienCell, Cat#0413) at 37 °C overnight and rinsed with Dulbecco’s phosphate-buffered saline (PBS; Gibco, Cat#14191-144) before use. Cryopreserved HAs were thawed at 37 °C, disinfected, and directly transferred into pre-coated flasks without centrifugation. The medium was replaced 16 hours after plating, then every 3 days until 70% confluency and every other day until 90% confluency. Cells were subcultured at 90–95% confluency using 0.05% trypsin/EDTA after washing with PBS. The cells were then neutralized, centrifuged, and replated at a density of 5000 cells/cm².

### Mice

Female C57BL/6J mice aged 9–13 weeks were purchased and maintained under conventional conditions with controlled temperature, humidity, and a 12-hour light/dark cycle. Animals had access to sterilized food and water *ad libitum* throughout the experiment. To accommodate the neurological deficits induced by EAE and the combined EAE-AQP4-IgG model, including reduced mobility and paralysis, HydroGel packs (ClearH2O, Cat#70-01-5022) and food pellets were placed directly on the cage floor to ensure that mice could easily access nutrition and hydration. At each designated endpoint, mice were deeply anesthetized using Avertin (tribromoethanol; Sigma-Aldrich, Cat#T48402) before tissue collection. Avertin stock solution was prepared by dissolving 2.5 g of 2,2,2-tribromoethanol in 1.55 mL of tert-amyl alcohol (DAEJUNG, Cat#1149-4100), stirring until fully dissolved, then diluting with sterile PBS (Hyclone, Cat#SH30256.01) to a final concentration of 100 mg/mL. The solution was filtered and stored in light-protected containers at 2–8 °C, with a shelf life of a day.

For temporal assessment, three independent endpoint cohorts were used, and mice were randomly allocated to the experimental groups included in each cohort using simple randomization. The day-13 cohort comprised 30 mice assigned to the WT, EAE, and AQP4-IgG groups (n = 10 per group) and was used for GFAP/C5b-9 double immunostaining and qRT-PCR. The day-21 cohort comprised 15 mice assigned to the WT, EAE, and AQP4-IgG groups (n = 5 per group) and was used for GFAP/AQP4 immunostaining, hematoxylin and eosin staining, and Luxol Fast Blue staining. The primary day-35 cohort comprised 40 mice assigned to the WT, EAE, AQP4-IgG-L, and AQP4-IgG-H groups (n = 10 per group). Clinical scores and survival were assessed in all 10 mice per group, and five mice per group were randomly selected for immunofluorescence, qRT-PCR, histological quantification, and transmission electron microscopy. Thus, a total of 85 mice were used across the three independent endpoint cohorts.

All animal procedures were approved by the Institutional Animal Care and Use Committee (IACUC) of Korea University (Approval No. KUIACUC-2022-0042).

### Purification and quantification of NMO-IgG

Blood from four NMOSD patients at Korea University Anam Hospital was collected and centrifuged to obtain plasma. NMO-IgG was purified from the plasma of anti-AQP4-IgG seropositive NMOSD patients using NAb™ Protein G Spin Columns (Thermo Scientific, Cat# 89960) following the manufacturer’s protocol. Plasma was diluted using binding buffer, loaded onto the column, washed, and eluted with IgG elution buffer. Eluates were then concentrated using Pierce™ Protein Concentrators (Thermo Scientific, Cat# 88540), and the IgG concentration was measured using a spectrophotometer (ThermoFisher Scientific, Cat# ND-ONE-W4). A final concentration of 40–200 µg/µL was used for the *in vivo* experiments.

Endpoint titers were not measured in our study. Instead, anti-AQP4 antibodies were evaluated using the EUROIMMUN cell-based indirect immunofluorescence assay, which provides semiquantitative results based on the visual intensity of specific immunofluorescence in AQP4-transfected cells. The scoring system is graded as 1+ (weak), 2+ (moderate), 3+ (strong), and 4+ (very strong). All four patients had a semiquantitative score of 2+, corresponding to moderate specific immunofluorescence.

All human biological sample usage was approved by the Institutional Review Board (IRB No. 2022AN0331).

### Induction of the combined EAE-AQP4-IgG model

EAE was induced via subcutaneous injection of 0.2 mL MOG35–55/CFA emulsion (Hooke Laboratories, Cat# EK-2110) at two sites: midline of upper and lower back (0.1 mL/site). Pertussis toxin (0.1 mL/dose) was administered intraperitoneally on the day of immunization and again 22–26 hours later. Mice were monitored daily for neurological symptoms; neurological disability was assessed daily using a standardized 0–5 clinical scoring system: 0, no clinical signs; 0.5, partial tail weakness; 1.0, limp tail; 1.5, limp tail with mild hindlimb weakness; 2.0, moderate hindlimb weakness; 2.5, unilateral hindlimb paralysis; 3.0, complete hindlimb paralysis; 3.5, hindlimb paralysis with partial forelimb weakness; 4.0, tetraplegia; 4.5, moribund state; 5.0, death or euthanasia due to disease severity. On days 8 and 11, 200 µg of purified NMO-IgG was administered intrathecally to the AQP4-L group, while 500 µg was administered to the AQP4-H group.

### Complement-dependent cytotoxicity assay

Primary human astrocytes (ScienCell) were seeded into 96-well plates at a density of 25,000 cells/well and cultured for 18–24 hours to reach confluency. Cells were washed with PBS (ScienCell) and incubated with heat-inactivated NMOSD patient-derived plasma (56 °C for 30 min) diluted in 50 μL of complete HA medium at 37 °C for 1 hour. After plasma incubation, cells were washed twice with PBS and subsequently incubated with 5% pooled human complement serum (Innovative Research, Cat#ICSER10ML) prepared in 50 μL of complete medium at 37 °C for 2 hours. Following complement treatment, cells were washed twice with PBS and incubated with 100 μL complete HA medium and 10 μL cell viability reagent (Invitrogen, Cat#DAL1025) at 37 °C for 1 h. Absorbance was measured using a microplate reader (Molecular Devices, Spectramax 340pc384) at 570 nm, with 600 nm used as the reference wavelength (normalized to the 600 nm value) to assess resorufin production as an indicator of cell viability. Cytotoxicity was calculated as a percentage by normalizing control wells with human astrocyte alone (defined as 0% cytotoxicity).

### Immunofluorescence staining

Brain, spinal cord, and optic nerve tissues were harvested and fixed in 4% paraformaldehyde at 4 °C overnight. Subsequently, tissues were dehydrated by sequential immersion in sucrose solutions at concentrations of 10% for 7 h, 15% for 4 h, and 30% for 12 h until fully equilibrated. After embedding in the OCT compound, the tissues were rapidly frozen in liquid nitrogen (–196 °C) and sectioned using a cryostat (Leica Biosystems, CM3050S) at thicknesses of 30 μm for the brain, 20 μm for the spinal cord, and 10 μm for the optic nerve. Tissue sections were rinsed 3 times in PBS and permeabilized and then blocked with a blocking buffer in PBS containing 1% bovine serum albumin (BSA; Bovogen, Cat#BSA100), 10% normal donkey serum (NDS; Sigma-Aldrich, Cat#S30, 100 mL) or normal goat serum (NGS; Sigma-Aldrich, S26, 100 mL), and 0.2% Tween-20 at room temperature for 1 hour. Sections were incubated at 4 °C overnight with the following primary antibodies diluted in blocking buffer: mouse anti-glial fibrillary acidic protein (GFAP; 1:500, Santa Cruz, sc-33673), rabbit anti-aquaporin 4 (AQP4; 1:400, Millipore, AB3594), rabbit anti-ionized calcium-binding adapter molecule 1 (Iba-1; 1:500, Wako, 019-19741), and anti-myelin basic protein (MBP; 1:200, Millipore, MAB386), as well as rat anti-mouse CD31 (1:200, BD Biosciences, 550274), rabbit anti-ZO-1 (1:200, Invitrogen, 61-7300), anti-mouse CD19 (1:100; BioLegend, Cat. #152407), and anti-C5b-9 (1:200; Abcam, Cat. #AB55811). After washing in PBS, sections were incubated with species-specific secondary antibodies conjugated to Alexa Fluor 594 or 488 (1:1000 in blocking buffer, Invitrogen) at room temperature for 1 hour. Nuclei were counterstained with Hoechst 33342 (1:2000 in PBS; Invitrogen, Cat#H3570) for 10 minutes. Slides were mounted using mounting medium (Vector Laboratories, Cat#H-1000-10) and imaged using a Leica fluorescence microscope DMB6 (Leica Microsystems) under consistent exposure settings. n = 5 mice per group were used for immunofluorescence analyses.

### Hematoxylin & eosin staining and inflammation scoring

Frozen tissue sections were rinsed in PBS and stained with Mayer’s hematoxylin solution (Abcam, Cat#AB220365) for 30 seconds. After washing in running tap water, the sections were differentiated in 1% acid alcohol for 1 minute, washed, and then immersed in a saturated lithium carbonate solution for 30 seconds for bluing. Subsequently, the sections were washed, treated with 95% ethanol for 30 seconds, and counterstained with eosin Y solution (Abcam, Cat#AB246824) for 30 seconds. Finally, the sections were dehydrated through a graded ethanol series (95%, 100%), cleared in xylene, and mounted using Permount™ mounting medium (ThermoFisher Scientific, SP15).

To assess inflammation severity, the stained sections were examined using a semi-quantitative scoring system. The degree of inflammation in brain and spinal cord tissues was graded on a scale of 0 to 3 as follows: 0, no inflammatory cells; 1, a few scattered inflammatory cells; 2, organization of inflammatory infiltrates around blood vessels; 3, extensive perivascular cuffing with extension into the parenchyma. For optic nerve sections, inflammation was graded on a scale of 0 to 4, where 0 indicated no infiltrating inflammatory cells and preservation of the bead-like arrangement of cell nuclei, and 4 indicated massive cellular infiltration. To ensure objectivity, all histological scoring and image quantification were performed by investigators blinded to the experimental group assignments. For each mouse, five randomly selected fields per section were evaluated, and the field-level scores were averaged to obtain one value per mouse.

### Luxol Fast Blue–hematoxylin & eosin co-staining

Frozen tissue sections were rinsed in PBS, then dehydrated through a graded ethanol series (70%, 80%, and 90%). Sections were incubated in 1% Luxol Fast Blue solution (Abcam, Cat#68260) at 60 °C for 1 hour to stain myelin sheaths. Following incubation, any excess staining solution was removed, and differentiation was performed sequentially using 0.05% lithium carbonate (Sigma-Aldrich, Cat#62470-100G-F) for 30–60 seconds, followed by 70% ethanol until an optimal gray-white matter contrast was achieved. After rinsing, sections were stained with Mayer’s hematoxylin solution (Abcam, Cat#AB220365) for 30 seconds, washed in running tap water, and briefly stained with eosin Y solution (Abcam, Cat#AB246824) for 30 seconds to visualize cellular and tissue structures. Sections were dehydrated through graded ethanol (95%, 100%) and cleared in xylene. Coverslips were mounted using Permount™ mounting medium (ThermoFisher Scientific, SP15). Stained sections were examined under bright-field microscopy to assess demyelination and inflammatory infiltration.

### qRT-PCR analysis

Total RNA was extracted from homogenized brain, spinal cord and optic nerve tissues using TRIzol reagent (Invitrogen, Cat#15596018) according to the manufacturer’s protocol. RNA concentration and purity were determined using Nanodrop One (ThermoFisher Scientific). A total of 5 μg of total RNA was reverse-transcribed into cDNA with oligo(dT) primers. Meanwhile, qRT-PCR was performed using SYBR Green PCR Master Mix (Enzynomic, RT501M) and analyzed on a quantitative real-time PCR machine (ThermoFisher Scientific, StepOnePlus). The primer sequences used in this study are listed in **Table 1**. For qRT-PCR analysis, tissues were collected from five mice per experimental group. For heatmap visualization, individual relative expression values were log2-transformed, and gene-wise Z-scores were calculated across all biological replicates within each tissue. The mean Z-score for each experimental group was then displayed in the heatmap.

**Table 1:** The primer sequences used for the quantitative RT-PCR analysis.

Gene (mouse)	Forward primer (5’→3’)	Reverse primer (5’→3’)
*GFAP*	ATG CCA CGC TTC TCC TTG TC	CTC CAT CAT CTC TGC ACG CT
*AQP4*	CTG GCA GGT GCC CTT TAT GA	AAG CTA TGT GGA GGT GCG AC
*MBP*	CAG AGT CCG ACG AGC TTC AG	CTA AAG AAG CGC CCG ATG GA
*OLIG2*	CTG GCG CGA AAC TAC ATC CT	CGT AGA TCT CGC TCA CCA GTC
*NG2*	TTA CCA TGC GGC CAA ATC CT	GGG ACG CTT CTT CCT GGT TT
*FGF17*	CGC CTG CTG CCT AAC CTT AC	TGG TCC CTC ACG TAC TGT GTT
*IL-1β*	GCC ACC TTT TGA CAG TGA TG	CGT CAC ACA CCA GCA GGT TA
*TNF-α*	CAC CGT CAG CCG ATT TGC TA	TTG GGC AGA TTG ACC TCA GC
*IL-6*	AGT TGC CTT CTT GGG ACT GA	TCC ACG ATT TCC CAG AGA AC
*CCL2*	AGG TGT CCC AAA GAA GCT GT	GAC CTT AGG GCA GAT GCA GTT
*PDGFα*	GGG TCC CAT GCC ATT AAC CA	ACT TGA CGC TGC TGG TGT TA
*PDGFβ*	AAC CTC ATC GAT CGC ACC AA	GGC ATT GCA CAT TGC GGT TA
*CNTF*	TTC GTT CAG ACC TGA CTG CTC	ACT CCA GCG ATC AGT GCT TG
*IL-17a*	CAG ACT ACC TCA ACC GTT CCA C	TCC AGC TTT CCC TCC GCA TTG A
*BCL6*	CAG AGA TGT GCC TCC ATA CTG C	CTC CTC AGA GAA ACG GCA GTC A
*PRDM1*	AAG ACG TTC GGT CAG CTC TCC A	CTG GCA CTC ATG TGG CTT CTC T
*CXCL13*	CAT AGA TCG GAT TCA AGT TAC GCC	GTA ACC ATT TGG CAC GAG GAT TC
*C1q*	TCA CCA ACC AGG AGA GTC CA	CCC TGG TAA ATG CGA CCC TT
*C3*	CCA GCT CCC CAT TAG CTC TG	GCA CTT GCC TCT TTA GGA AGT C
*C5*	AAC ACC CTG CCA TCC AAG AG	CAC AGA GCT GTC TGG ACG TT
*C5aR1*	ATG GCA TTC ACC TCC CGA AG	ACT GGC GTA CAT GTT GAG CA
*GAPDH*	TGA CAT CAA GAA GGT GGT GAA GC	CCC TGT TGC TGT AGC CGT ATTC

### TEM

Mice were anesthetized using an Avertin solution and perfused transcardially with PBS, followed by fixation using a solution of 2% paraformaldehyde (Electron Microscopy Sciences, Cat#15700-5G) and 2% glutaraldehyde (Electron Microscopy Sciences, Cat#16130) in 0.2 M sodium cacodylate buffer (Electron Microscopy Sciences, Cat#11653). The brain, spinal cord, and optic nerve tissues were dissected and then fixed in the same fixative at 4 °C overnight. After washing in buffer, samples were postfixed in 2% aqueous osmium tetroxide (Electron Microscopy Sciences, Cat#19152) at room temperature for 1 hour, dehydrated through a graded ethanol series, infiltrated with propylene oxide, and embedded in epoxy resin. Ultrathin sections were stained with lead citrate and examined using a transmission electron microscope (JEM1010, JEOL, Japan) operated at 80 kV. For transmission electron microscopy analysis, tissues were obtained from five mice per experimental group.

### Western blot analysis

Patient-derived IgG was purified from the peripheral blood of four patients with NMOSD. Purified IgG samples were mixed with non-reducing lane marker sample buffer (Thermo Fisher Scientific, Cat#39001) and distilled water, boiled, and 10 ng of NMO-IgG per sample was loaded per lane. Proteins were separated by SDS–PAGE at 120 V for 1.5 hours and transferred onto nitrocellulose membranes (NC membrane; Cytiva, Cat#10600002) at 100 V for 2 hours. Membranes were blocked with 5% skim milk in phosphate-buffered saline containing 0.1% Tween-20 (PBST) at room temperature for 1 hour and incubated overnight at 4 °C with a primary antibody against human IgG (anti-human IgG; 1:5000, Abcam, Cat#ab109489) diluted in 5% bovine serum albumin (BSA) in PBST. After washing with PBST, membranes were incubated with an HRP-conjugated secondary antibody (1:10000, Santa Cruz Biotechnology, Cat#sc-2354) at room temperature for 1 hour. Protein bands were visualized using SuperSignal™ West Femto Maximum Sensitivity Substrate (Thermo Fisher Scientific, Cat# PI34095) and imaged using a ChemiDoc imaging system with VisionWorks software.

### Statistical analysis

Fluorescence intensity and marker-positive area were quantified using ImageJ software by investigators blinded to the experimental group assignments; image files were coded prior to analysis and decoded only after quantification was complete. Statistical analyses were conducted using IBM SPSS Statistics. Levene’s test was first used to assess homogeneity of variance. If equal variance was assumed, one-way analysis of variance (ANOVA) followed by Bonferroni *post hoc* analysis was conducted. If unequal variance was detected, Welch’s ANOVA was used instead, followed by the Games–Howell *post hoc* test. Sample sizes were determined based on prior published studies using similar rodent models of NMOSD and EAE (21, 23, 39), in which n = 5 per group has been shown to provide sufficient statistical power to detect biologically meaningful differences in histological and molecular analyses. Formal *a priori* power analysis was not performed; however, the observed effect sizes in key outcome measures (GFAP, AQP4, LFB staining, and qRT-PCR) were large, and statistically significant differences were consistently detected across all three CNS regions with n = 5 per group, supporting the adequacy of the chosen sample size. An asterisk (*) in the figures and tables denotes statistical significance compared to the WT group, while a hash symbol (#) indicates significance compared to the EAE group. The number of symbols corresponds to the level of significance: * or # for *P* < 0.05, ** or ## for *P* < 0.01, and *** or ### for *P* < 0.001.

## Discussion

This study establishes and validates a combined EAE-AQP4-IgG model through intrathecal administration of patient-derived NMO-IgG and human complement into pre-immunized EAE mice. Whereas previous antibody–complement-based NMOSD models largely aimed to reproduce astrocyte loss and complement-mediated cytotoxicity, our model suggests that astrocytopathy is not only a pathological outcome but also an important organizer of CNS inflammatory architecture. Indeed, AQP4-IgG-induced astrocyte injury in our model system appears to redirect the immune landscape, converting the CCL2-dominant, repair-permissive inflammatory environment characteristic of EAE into an IL-6–CXCL13–PRDM1-driven, B cell-amplifying, remyelination-inefficient state that closely resembles the immunopathological context of human NMOSD.

The divergent astrocytic states between the combined EAE-AQP4-IgG model and EAE — destruction versus reactive astrogliosis - were associated with fundamentally different inflammatory architectures, supporting the concept that astrocyte integrity is a central determinant of the CNS immune microenvironment. These observations support the idea that astrocyte injury in NMOSD may be not merely a passive consequence of inflammation but an initiating event that destabilizes tissue homeostasis and contributes to secondary immune reprogramming.

BBB disruption in NMOSD revealed a fundamental mechanistic distinction from EAE. Whereas BBB permeability in EAE arises largely from cytokine-mediated inflammation and T cell trafficking, NMOSD involves direct astrocyte–endothelial injury initiated by AQP4-IgG and complement activation (40, 41), resulting in structural disintegration of the astrocyte–endothelial unit rather than merely reversible barrier leakage. This process may contribute to irreversible astrocyte loss, sustained vascular leakage, and widespread demyelination, consistent with the acute necrotic lesions observed in NMOSD.

The higher expression of *IL-6*, *IL-17A*, and *CXCL13*, together with the *PRDM1*-high/*BCL6*-low transcriptional pattern observed in the combined model relative to EAE, is consistent with an enhanced Th17- and B-cell-associated inflammatory profile superimposed on EAE-associated neuroinflammation (29, 30, 36, 42, 43). Importantly, the increased IL-6 and CXCL13 expression observed at the transcriptional level was further supported by immunofluorescence analysis, which demonstrated increased IL-6- and CXCL13-positive areas in the brain, spinal cord, and optic nerve of the combined EAE-AQP4-IgG model relative to EAE (**Supplementary Figure 3**). This distinction directly aligns with clinical success of IL-6 blockade, such as tocilizumab and satralizumab, and supports evaluation of B cell-derived therapies (44).

These findings indicate that astrocytopathy extends beyond glial injury to influence the entire cellular network responsible for remyelination, as the loss of astrocyte-derived trophic support creates a fundamentally non-permissive environment for OPC survival and differentiation (14, 35, 45). This mirrors the clinical irreversibility of NMOSD demyelination in contrast to the partial remyelination capacity observed during MS remission (1, 46), underscoring the disease-specific consequences of astrocyte destruction on CNS regenerative potential.

The reciprocal relationship between astrocyte destruction and microglial activation supports a model in which astrocytopathy propagates demyelination through local cytokine release, oxidative stress, and phagocytic signaling (47, 48), collectively delineating a distinct pathogenic cascade for NMOSD initiated by humoral immune attacks on astrocytes and amplified by innate immune responses.

By employing intrathecal delivery of patient-derived NMO-IgG in a pre-immunized background, our model overcomes these limitations of prior systems restricted by an intact BBB (49), enabling direct, region-specific exposure of CNS astrocytes to pathogenic antibodies and exogenous human complement. The resulting astrocytopathy and demyelination patterns share key features with human NMOSD lesions described in clinical neuropathology (24, 36). Therefore, this optimized model provides a powerful platform for understanding the mechanisms underlying astrocyte injury, inflammatory amplification, and remyelination failure, and serves as a preclinical system for evaluating therapeutic strategies targeting complement activation, cytokine signaling, astrocyte protection, or BBB restoration. The ability of this model to reproduce optic nerve and spinal cord pathology makes our model especially valuable for studying visual and motor impairments characteristic of NMOSD.

While our model successfully reproduces the major pathological hallmarks of NMOSD, several limitations should be considered. First, the model is established on an EAE-pre-immunized background and thus represents a combined EAE-AQP4-IgG model rather than a pure NMOSD model; the residual T cell-mediated inflammatory components inherent to EAE cannot be fully excluded. The expression levels of *IL-17A* and *CXCL13* were increased in EAE relative to WT and were further elevated in the combined model relative to EAE. However, because an AQP4-IgG/human-complement-only group was not included, these additional increases cannot be attributed exclusively to antibody/complement-mediated astrocytopathy. Future studies employing AQP4-IgG and human complement without EAE pre-immunization will be required to isolate their specific contribution. Second, intrathecal delivery of patient-derived AQP4-IgG and human complement bypasses systemic immune processes such as peripheral B cell activation and antibody trafficking, limiting translational fidelity. Third, although temporal analyses at Day 13, Day 21, and Day 35 revealed a progressive pathological cascade — from early complement activation through astrocyte loss and neuroinflammation to established demyelination — the model does not yet capture the chronic, relapsing course typical of human NMOSD. Future studies incorporating later timepoints or relapse-induction protocols will be needed to fully recapitulate the long-term disease trajectory. Finally, reliance on human complement in a rodent system may generate interspecies differences in effector function. Hence, future refinements will be needed to reproduce the chronic, relapsing nature of NMOSD pathology more accurately. Additionally, the inflammatory and regenerative pathways described in this study are primarily inferred from qRT-PCR-based mRNA expression data. Although the observed transcriptional signatures are corroborated by immunostaining evidence of complement deposition (C5b-9) and B cell infiltration (CD19), and are consistent with protein-level findings reported in NMOSD patient CSF in prior clinical studies (33, 42, 50, 51), the increased expression of IL-6 and CXCL13 was further validated at the protein level by immunofluorescence in the brain, spinal cord, and optic nerve (**Supplementary Figure 3**). Future studies employing ELISA, Western blotting, or flow cytometry for key molecules such as IL-17A, PRDM1, and BCL6 would provide additional validation of the proposed immune architecture.

In conclusion, the combined EAE-AQP4-IgG model developed in this study addresses a critical gap between experimental and clinical research by recapitulating key aspects of astrocytopathy-driven demyelination and neuroinflammation, which are mechanistically distinguishable from MS pathology. Comparative analyses with EAE suggest that the pathogenesis of NMOSD is largely mediated by antibody-dependent astrocyte destruction, associated with demyelination. In contrast, MS is characterized by T cell-driven immune infiltration with partial regenerative potential. This distinction underscores the necessity for disease-specific therapeutic strategies. Overall, this combined EAE-AQP4-IgG model provides a robust experimental platform for elucidating the antibody-mediated pathogenic mechanisms of NMOSD and advancing targeted treatments.

## Data Availability

The original contributions presented in the study are included in the article/**Supplementary Material**. Further inquiries can be directed to the corresponding authors.
