# Supplementary material for: AQP4 antibody-induced astrocyte injury reshapes CNS immune architecture in a combined EAE–AQP4-IgG model of neuromyelitis optica spectrum disorder
Source: Front Immunol. 2026 Oct 19;17:1899823. doi: 10.3389/fimmu.2026.1899823 (PMC13724468; doi:10.3389/fimmu.2026.1899823)
Supplement: Supplementary file 1 [file DataSheet1.docx]

Supplementary Material

## Supplementary Figures

**Supplementary Fig. 1. Complement-dependent cytotoxicity (CDC) assay using patient-derived NMO-IgG and human complement.**

(A) Schematic overview of the CDC assay. Human astrocytes (HAs) were incubated with patient-derived anti-AQP4 antibodies and human complement and then assessed for complement-dependent cytotoxicity using the alamarBlue assay. (B) Western blot analysis confirmed the presence of human IgG in patient-derived neuromyelitis optica immunoglobulin G antibody (NMO-IgG) samples used for the CDC assay. Western blotting was performed to confirm the presence and integrity of patient-derived NMO-IgG prior to in vitro and in vivo experiments. (C) Quantitative analysis of CDC activity in astrocyte cultures treated with sera from four NMOSD patients. An increase in serum concentrations led to enhanced cytotoxicity, indicating a dose-dependent astrocyte injury mediated by anti-AQP4 antibodies and complement activation.

**Supplementary Fig. 2. Distinct patterns of microglial activation and myelin loss in EAE-AQP4-IgG model compared to the EAE model at day 35.**

(A, D, G) Representative immunofluorescence images of MBP and Iba1 in the brain (A), spinal cord (D), and optic nerve (G) from WT, EAE, and ENC mice at day 35. Both EAE and ENC tissues exhibited reduced MBP immunoreactivity and increased Iba1-positive microglial accumulation compared with WT tissues, with more pronounced changes in ENC mice. (B, E, H) Quantification of Iba1-positive cells in the brain (B), spinal cord (E), and optic nerve (H) showed significantly greater microglial accumulation in EAE-AQP4-IgG mice than in EAE mice. (C, F, I) Quantification of MBP fluorescence intensity in the brain (C), spinal cord (F), and optic nerve (I) showed significantly lower MBP immunoreactivity in EAE-AQP4-IgG mice than in EAE mice. Five mice were analyzed per group. Data are presented as mean ± SD. *P < 0.05, **P < 0.01, and ***P < 0.001 versus WT; #P < 0.05, ##P < 0.01, and ###P < 0.001 versus EAE. Scale bars are indicated in the images.

**Supplementary Figure 3. Increased IL-6 and CXCL13 protein expression in the combined EAE-AQP4-IgG model.**

(A, D, G) Representative immunofluorescence images of IL-6 (green), CXCL13 (red), and Hoechst (blue) in the brain (A), spinal cord (D), and optic nerve (G) of WT, EAE, and combined EAE-AQP4-IgG model mice. (B, E, H) Quantification of IL-6-positive area per ROI in the brain (B), spinal cord (E), and optic nerve (H). (C, F, I) Quantification of CXCL13-positive area per ROI in the brain (C), spinal cord (F), and optic nerve (I). Data are presented as mean ± SD; *P < 0.05, **P < 0.01, ***P < 0.001 vs. WT; #P < 0.05, ##P < 0.01, ###P < 0.001 vs. EAE. Scale bar, 20 μm.

**Supplementary Fig. 4. Regional gene expression profiles in the brain, spinal cord, and optic nerve.**

(A–C) Relative mRNA expression of complement-related genes (C1q, C3, C5, and C5aR1) in the brain (A), spinal cord (B), and optic nerve (C). (D–F) Relative mRNA expression of inflammatory cytokine- and chemokine-related genes (IL-6, CCL2, TNF-α, IL-1β, and IL-17A) in the brain (D), spinal cord (E), and optic nerve (F). (G–I) Relative mRNA expression of B cell-related genes (CXCL13, PRDM1, and BCL6) in the brain (G), spinal cord (H), and optic nerve (I). (J–L) Relative mRNA expression of myelin- and oligodendrocyte precursor cell (OPC)-related genes (MBP, Olig2, NG2, and FGF17) in the brain (J), spinal cord (K), and optic nerve (L). (M–O) Relative mRNA expression of astrocyte-derived trophic factor genes (PDGFα, PDGFβ, and CNTF) in the brain (M), spinal cord (N), and optic nerve (O). Data are presented as mean ± SD. P < 0.05, P < 0.01, and P < 0.001 vs. WT; #P < 0.05, ##P < 0.01, and ###P < 0.001 vs. EAE.
